# Spatiotemporal Dynamics and Trend Analysis of African Swine Fever Virus in Romanian Wild Boar Populations (2018–2024)

**DOI:** 10.3390/ani16203202

**Published:** 2026-10-12

**Authors:** Corina Pascu, Corina Badea, Viorel Herman, Kalman Imre, Florica Bărbuceanu, Oana Tănase, Luminița Costinar

**Affiliations:** 1 Department of Infectious Diseases and Preventive Medicine, Faculty of Veterinary Medicine, University of Life Sciences “King Mihai I”, 300645 Timisoara, Romania; corinapascu@usvt.ro (C.P.); viorel.herman@fmvt.ro (V.H.); luminita.costinar@usvt.ro (L.C.); 2 Department of Food Safety and Hygiene, Faculty of Veterinary Medicine, University of Life Sciences “King Mihai I”, 300645 Timisoara, Romania; kalmanimre@usvt.ro; 3 Institute for Diagnosis and Animal Health (IDAH), 050557 Bucharest, Romania; florica.barbuceanu@idah.ro; 4 Faculty of Veterinary Medicine, University of Agronomical Sciences and Veterinary Medicine, 050097 Bucharest, Romania; 5 Department of Public Health, “Ion Ionescu de la Brad” Iasi University of Life Sciences, 8 Sadoveanu Alley, 707027 Iasi, Romania; oana.tanase@iuls.ro

**Keywords:** African Swine Fever, wild boar, spatiotemporal trend, SatScan, Generalized Additive Models (GAMs), epidemiology, Romania

## Abstract

**Background:** African Swine Fever (ASF) remains a critical threat to the swine industry globally, with wild boars (*Sus scrofa*) acting as a major wildlife reservoir. Romania has been one of the most severely affected European countries since the virus’s introduction. **Objectives:** This study aims to estimate the long-term spatiotemporal trends, environmental persistence, and transmission dynamics of ASF virus (ASFV) in the Romanian wild boar population based on a finalized dataset of 1443 confirmed cases distributed across an 80-month timeline (May 2018–December 2024). **Methods:** Advanced epidemiological workflows integrating spatial and temporal modeling frameworks were developed. Spatiotemporal cluster detection was performed using SatScan to identify significant disease hotspots. Generalized Additive Models (GAMs) were utilized to model non-linear seasonal trends and spatial patterns of carcass persistence. Furthermore, the time-varying reproduction number (*R_t_*) was calculated using a Bayesian renewal framework via the EpiEstim package, parameterized with a wild boar serial distribution (mean = 10.3 days; SD = 4.2 days). **Results:** SatScan identified one most primary cluster in Dobrogea and five other major recurrent hot spots in Muntenia, Transylvania, and Moldova. The GAM analysis revealed a strong, statistically significant seasonal pattern, with case detection from carcasses peaking markedly during the winter months when low temperatures favor environmental virus stability. The Bayesian *R_t_* reconstruction disclosed three distinct epidemiological phases: a sharp initial invasion peak at a mean *R_t_* of 2.38 (95% Crl: 1.82–3.02), followed by regional wave-like oscillations, and a distinct long-term stabilization in the late endemic phase, hovering dynamically around a stationary mean of 1.01 (95% Crl: 0.87–1.16). **Conclusions:** Our findings statistically validate the shift from an acute epidemic wave to a self-sustaining, chronic endemic equilibrium within the natural habitat, driven by constant carcass-mediated environmental re-seeding. These insights underscore that successful disease mitigation must prioritize the rapid, targeted removal of infected carcasses in high-risk forest matrices rather than generic population reduction strategies.

## Simple Summary

African Swine Fever is a severe viral disease that threatens global pig farming, with wild boars serving as a persistent natural reservoir, keeping the virus alive in the environment. This study aimed to track how the virus spread and changed across Romania between 2018 and 2024 to help improve disease management. The analysis revealed that the epidemic expanded slowly but steadily over 73 months, moving in waves from southeastern regions like Dobrogea into central and northern parts of the country. Crucially, virus detection peaked in winter, as cold temperatures prevent the virus from breaking down, allowing carcasses to contaminate the environment over the long term. Over time, the virus reached a stable state, meaning it is now permanently established in local wildlife ecosystems rather than causing temporary outbreaks. This study is highly valuable to society because it shows that stopping the disease requires moving away from simply reducing the overall wild boar population. Instead, authorities must focus on fast, targeted removal of dead boars in high-risk forests to protect the livestock industry and food security.

## 1. Introduction

African Swine Fever (ASF) is a highly contagious and lethal viral disease affecting domestic and wild suids, caused by a double-stranded DNA virus belonging to the *Asfarviridae* family [1,2]. In the European Union, the disease has generated devastating economic and agricultural consequences, notably disrupting the swine sector [3,4]. Romania recorded its first ASF outbreak in July 2017 in a backyard holding within Satu Mare County, following a rapid geographical expansion from neighboring Ukraine and the Republic of Moldova. Since its introduction, the virus has quickly become endemic across the entire Romanian territory. The epidemiological landscape in Romania is uniquely characterized by a massive dual presence of the virus: it simultaneously impacts a vast network of low-biosecurity backyard farms and free-ranging wild boar populations (*Sus scrofa*) [5,6,7,8]. This complex transmission interface has facilitated the emergence of distinct epidemic waves, with surveillance data confirming approximately 3000 cases between 2017 and mid-2019. Subsequent surveillance efforts have sought to quantify the front-wave velocity of the infection, providing essential insights into the virus’s transmission dynamics across diverse European habitats [9,10,11,12]. Despite these advancements, persistent epidemiological challenges remain, particularly regarding the role of wild boar in the maintenance of the virus throughout the European Union [13,14]. To address these challenges, researchers have increasingly relied on spatiotemporal modeling and cluster analysis to delineate the disease’s movement across diverse habitats [15]. Such quantitative investigations are crucial for distinguishing between stochastic local outbreaks and sustained regional spread, as evidenced by the varied spatial patterns of persistence observed across different European landscapes [16]. These diverse spatial dynamics have led to the development of sophisticated Bayesian hierarchical frameworks, which integrate landscape features and host abundance to refine the predictive accuracy of carcass detection efforts [17]. Furthermore, while some regions have successfully contained the pathogen through targeted management of wild boar populations, others have faced challenges due to the transboundary movement of infected animals [18]. Moreover, while human-mediated long-distance dispersal—often linked to the improper disposal of contaminated food products—frequently precipitates new, isolated outbreaks, the subsequent persistence of the virus within local wild populations remains a primary driver of endemicity [19,20]. To disentangle these multifaceted transmission routes, researchers have employed machine learning techniques, such as random forest algorithms, to evaluate how environmental, demographic, and agricultural variables influence the persistence of these regional clusters [6].

Wild boars serve as a primary wildlife reservoir that secures the long-term persistence and transmission of the ASF virus (ASFV) in the natural environment [21,22]. Epizoo tiological modeling calibrated on Romanian data demonstrates that ASFV transmission at the wildlife–livestock interface is strongly bidirectional. While inter-farm movements drive domestic outbreaks, approximately 68% of infections in pig farms originate from nearby wild boar populations [23,24], whereas wild boars frequently acquire the virus through spillover events from infected backyard herds. Beyond these direct contact dynamics, the virus’s environmental stability in carcasses and its capacity for persistence within regional forest habitats further complicate control efforts [25]. Specifically, carcass-based transmission has been estimated to account for a substantial proportion of infections, particularly in low-density populations where direct contact rates are insufficient to sustain the pathogen [24]. Consequently, effective mitigation necessitates a dual approach that integrates rigorous population density reduction with the prompt removal of wild boar carcasses to eliminate these long-term environmental reservoirs [26]. Moreover, implementing landscape-level fencing and movement restrictions, combined with enhanced on-farm biosecurity, remains vital to minimizing the interspecific interactions that facilitate these recurring spillover events [27,28].

Consequently, the persistence of the pathogen is further exacerbated by human-mediated factors, including the illegal movement of contaminated pork products and the lack of stringent biosecurity measures in small-scale holdings [7,8]. The situation is further complicated by the country’s high density of non-commercial holdings, which accounted for over 800,000 units at the onset of the epidemic. The significant discrepancy between official registry data and the actual number of animals further complicates management, as millions of backyard pigs remain unregistered and outside the scope of standardized disease control protocols. Furthermore, the environmental stability of the virus in carcasses and fomites necessitates rigorous carcass disposal and sanitation protocols to mitigate the risk of spillover at this permeable interface.

In the wild boar habitat cycle, ASFV’s extreme environmental resistance facilitates continuous transmission. Wild boars are highly susceptible to the virus, experiencing mortality rates close to 90–100% in acute cases. The highly stable nature of the virus allows it to persist for months in unrecovered carcasses, enabling environmental transmission to healthy individuals even when local population densities drop. This “wild boar–habitat cycle” creates a self-sustaining reservoir that functions independently of direct animal-to-animal contact, complicating traditional culling strategies [1]. Furthermore, the potential emergence of ‘survivor’ individuals—those that recover from initial infection yet retain the capacity to re-excrete the virus during subsequent viraemic phases—may fundamentally alter long-term population dynamics [29].

Furthermore, dense forest cover and vast agricultural lands provide ideal habitats and foraging grounds for wild boars, particularly in expansive wetland zones like the Danube Delta. The cultivation of crops adjacent to smallholdings attracts wild boars, significantly increasing the probability of indirect contact through contaminated forage, soil, or fomites [1,22,30]. The movement patterns of wild boars introduce a seasonal velocity to the spread of the virus. During the summer crop-foraging season and the autumn breeding season, increased movements across hunting grounds and international borders trigger wave-like transmission patterns that actively spread the disease from east to west. These transboundary migrations are compounded by anthropogenic factors, as longer-distance viral translocations are frequently attributed to the illegal movement of infected carcasses or pork products across regional and national borders [31]. Such anthropogenic influences necessitate enhanced cross-border cooperation in surveillance strategies, as the synchronization of monitoring efforts remains the only viable mechanism to counteract the epidemic’s expansion [32].

The present study aimed to investigate the spatiotemporal dynamics of ASF in wild boar in Romania between 2018 and 2024 and to assess their role in virus persistence based on data from the national surveillance program. By analyzing case reports, the study sought to identify patterns of spread, persistence, and potential determining factors that influence the epidemiology of ASF in wild boar. Understanding these dynamics is essential for informing targeted interventions and improving disease management strategies. Data on ASF cases in wild boar reported through the national surveillance program between 2018 and 2024 were analyzed. Descriptive epidemiological methods were applied to assess temporal trends. Spatiotemporal models were examined to identify clusters and areas of infection persistence. Advanced statistical techniques, including spatiotemporal scan statistics, were used to detect significant clusters and recurrent hotspots, enabling accurate interpretation of ASF distribution over time and space. The results highlighted sustained circulation of the ASF virus in wild boar populations throughout the study period, with marked spatial heterogeneity. These recurrent hotspots were predominantly identified in border regions, suggesting that transboundary transmission dynamics play a critical role in the maintenance of viral foci [33]. Additionally, the observed seasonal peaks in these hotspots frequently align with periods of increased wild boar activity, suggesting that environmental factors and population movement patterns significantly influence transmission intensity [34,35]. These findings indicate that habitat connectivity and anthropogenic land-use patterns exacerbate the formation of these infection corridors, necessitating a refined spatial risk model to prioritize surveillance in areas identified as high-impact environmental interfaces [36,37]. Empirical evidence consistently demonstrates that passive surveillance, prioritizing the testing of wild boar found dead, serves as a significantly more sensitive diagnostic approach for detecting the pathogen compared to traditional active surveillance methods [38,39]. This increased sensitivity is critical, as active hunting strategies alone often prove insufficient for eradicating the virus from the landscape without the concurrent implementation of systematic carcass removal [40]. Areas of high incidence and recurrent clusters were identified, suggesting localized persistence of the virus in certain geographic regions. Temporal analysis indicated seasonal fluctuations in case detection, possibly associated with variations in surveillance intensity, wild boar behavior, and ecological factors such as food availability and climatic conditions. The persistence of ASF in wild boar appears to be determined by a combination of population density, environmental conditions, and the limited effectiveness of control measures, highlighting the challenges in interrupting virus transmission. These findings emphasize the necessity of prioritizing passive surveillance efforts, as data consistently show a higher detection rate in animals found dead compared to those harvested through active hunting. Moreover, preventive strategies focused on stabilizing wild boar densities before viral introduction remain essential for reducing the probability of population exposure and minimizing the resource demands of future emergency responses [19,35]. Wild boars play a key role in the long-term persistence of ASF in Romania, acting both as a reservoir and a source of infection [19,41]. The identified spatiotemporal patterns underscore the need for region-specific targeted control strategies and enhanced surveillance. These strategies should integrate ecological and behavioral factors that influence wild boar populations to increase the effectiveness of interventions [20,27,36,42,43]. Accordingly, surveillance and control measures should prioritize recurrent hotspots and high-risk environmental interfaces rather than applying uniform interventions across all regions. A more comprehensive understanding of ASF dynamics in wild boar is therefore essential for developing effective disease-management and eradication programs. These programs must reconcile ecological constraints with epidemiological imperatives and combine sustained surveillance, targeted interventions, and adaptive management to achieve durable reductions in transmission and sustainable disease control [19,29,44,45,46]. Furthermore, the establishment of low-density buffer zones through female-targeted hunting and strictly enforced feeding bans may serve as a critical long-term measure to limit viral spread across these identified environmental interfaces [47]. Additionally, refining carcass search protocols through spatial predictive modeling could optimize resource allocation in these zones, as identifying predilection sites significantly enhances the efficacy of passive monitoring [48].

## 2. Materials and Methods

### 2.1. Data and Software Used

Cases of African Swine Fever (ASF) in wild boar in Romania during the period May 2018–December 2024 were analyzed, encompassing a finalized dataset of 1443 confirmed cases distributed across an 80-month timeline. This information was obtained from the databases of ANSVSA, EFSA, and WAHID [49,50,51]. Each record represented a laboratory-confirmed case of African Swine Fever and included the following variables: year, case (carcass/hunted wild boar), latitude, longitude, and date of confirmation. Each entry was considered a single epidemiological unit (one infected animal or carcass). The data were thoroughly checked to remove missing values and obvious recording errors. To evaluate temporal and spatial dynamics, data were processed in Python (v.3.11) using the pandas package (v2.2) for data management and aggregation, and numpy (v1.26) for numerical computation. Advanced spatiotemporal scan statistics and cluster identification were performed using SatScan (v10.3.3) under the Space–Time Permutation model guidelines. Part of the coding structures and language editing in R environment were supported by the AI-based language model, using ChatGPT 5.0. All advanced mathematical modeling, including non-linear seasonal trends and the time-varying reproduction number (*R_t_*) estimations, were performed in the R programming environment (v4.5.3) using the mgcv, EpiEstim, and ggplot2 packages. For the transmission intensity analysis, individual case entries were converted to ISO format (YYYY-MM-DD) and aggregated at a daily incidence resolution to satisfy the renewal equation operational requirements.

### 2.2. Temporal Analysis and Transmission Dynamics

To systematically evaluate the temporal evolution of the epidemic, the daily incidence of confirmed African Swine Fever (ASF) cases was utilized to construct the core epidemic curve across the 80-month study period. To evaluate transmission intensity and verify the progression toward endemic stabilization, the time-varying reproduction number (*R_t_*) was estimated using a non-parametric Bayesian framework implemented via the EpiEstim package in R. Unlike simple empirical growth ratios, this method computes *R_t_* as the ratio of the number of new infections at time t to the cumulative infectivity of all previously infected animals, modulated by the serial interval distribution:

$$R_{t}=\frac{I_{t}}{\sum_{s=1}^{t}I_{t-s}w_{s}}$$

where *I_t_* represents the daily case incidence at day *t* and *w_s_* is the probability density function of the serial interval (SI) at day *s*. The serial interval distribution was parameterized using a continuous parametric Gamma distribution with a mean of 10.3. days and a standard deviation (SD) of 4.2 days, derived from established European wildlife epidemiological consensus [52] and reflecting the biological transmission cycle of African Swine Fever Virus (ASFV) within wild boar populations under field conditions. To minimize statistical noise caused by daily reporting delays while preserving the ability to capture rapid changes in transmission trends, *R_t_* estimates and their corresponding 95% Bayesian credible intervals (Crl) were calculated using 7-day sliding weekly windows. An *R_t_* mean statistically higher than 1.0 indicates progressive epidemic growth, while an *R_t_* converging and stabilizing around 1.0 mathematically confirms a stationary endemic state sustained within the wildlife reservoir.

### 2.3. Spatiotemporal Analysis

To identify statistically significant spatiotemporal clusters of African Swine Fever (ASF) cases in wild boar in Romania, SaTScan version 10.3.3, was utilized. SaTScan™ is a registered trademark of Martin Kulldorff and was developed in collaboration with the National Cancer Institute and the New York City Department of Health and Mental Hygiene, providing a well-established and widely recognized platform for complex epidemiological analyses [53]. Spatiotemporal analysis was conducted using the Space–Time Permutation model, which is appropriate when only case data are available and comprehensive information on the underlying population at risk is lacking [8,18]. The analysis analyzed the finalized dataset of 1443 laboratory-confirmed cases of ASF in wild boar, georeferenced at 1365 distinct locations across Romania, spanning the entire 80-month timeline. The analysis was executed at a daily temporal resolution to capture precise outbreak onset profiles. Spatial and temporal bounding windows were established, with, a maximum spatial window corresponding to 15% of the population at risk and a maximum temporal window threshold of 180 days to optimize cluster detection sensitivity while preventing over-segmentation [54]. Statistical significance of the identified clusters was evaluated using 999 Monte Carlo replications to estimate the probability of cluster occurrence under the null hypothesis of a completely random statiotemporal distribution. Clusters were considered statistically significant if the *p*-value was less than 0.05, establishing a consistent empirical framework for tracing localized ASF persistence and propagation trajectories across the country.

### 2.4. GAM (Generalized Additive Model) for Determining the Temporal Trend of ASF Virus Persistence

To model the temporal trend and ecological patterns of ASFV persistence in the natural habitat, a non-linear Generalized Additive Model (GAM) with a binomial error distribution was applied to the monthly proportion of carcasses relative to the total reported cases (carcasses found dead/[carcasses + hunted animals]). This modeling approach allows the evaluation of non-linear intra-annual seasonal components and long-term multi-year variations without forcing a rigid parametric linear constraint. The analytical workflow involved the complete aggregation of the monthly metrics across the 80-month window, computing the carcass-to-total ratio as a structural indicator of environmental virus stability and passive surveillance intensity. The binomial GAM incorporated cubic regression splines to model temporal components flexible enough to isolate cyclic seasonality from the absolute multi-year trajectory. Continuous visualizations were generated incorporating the estimated smooth curve fits accompanied by their corresponding 95% confidence intervals (CI). Epidemiological trends were classified based on the smooth term significance test results; a *p*-value < 0.05 indicated a statistically significant systematic shift in the environmental carcass deposition pattern, whereas a *p*-value ≥ 0.05 signified that the dynamic was predominantly driven by recurring stationary seasonal oscillations.

To complement the spatial component of environmental virus stability, a localized geographic persistence index (PI) was calculated using the formula:

$$\mathrm{PI}=\;\frac{\mathrm{the}\;\mathrm{number}\;\mathrm{of}\;\mathrm{years}\;\mathrm{with}\;\mathrm{at}\;\mathrm{least}\;\mathrm{one}\;\mathrm{case}}{7}$$

The interpretation of this index was carried out according to Table 1.

## 3. Results

### 3.1. Temporal Dynamics

**Figure 1:**
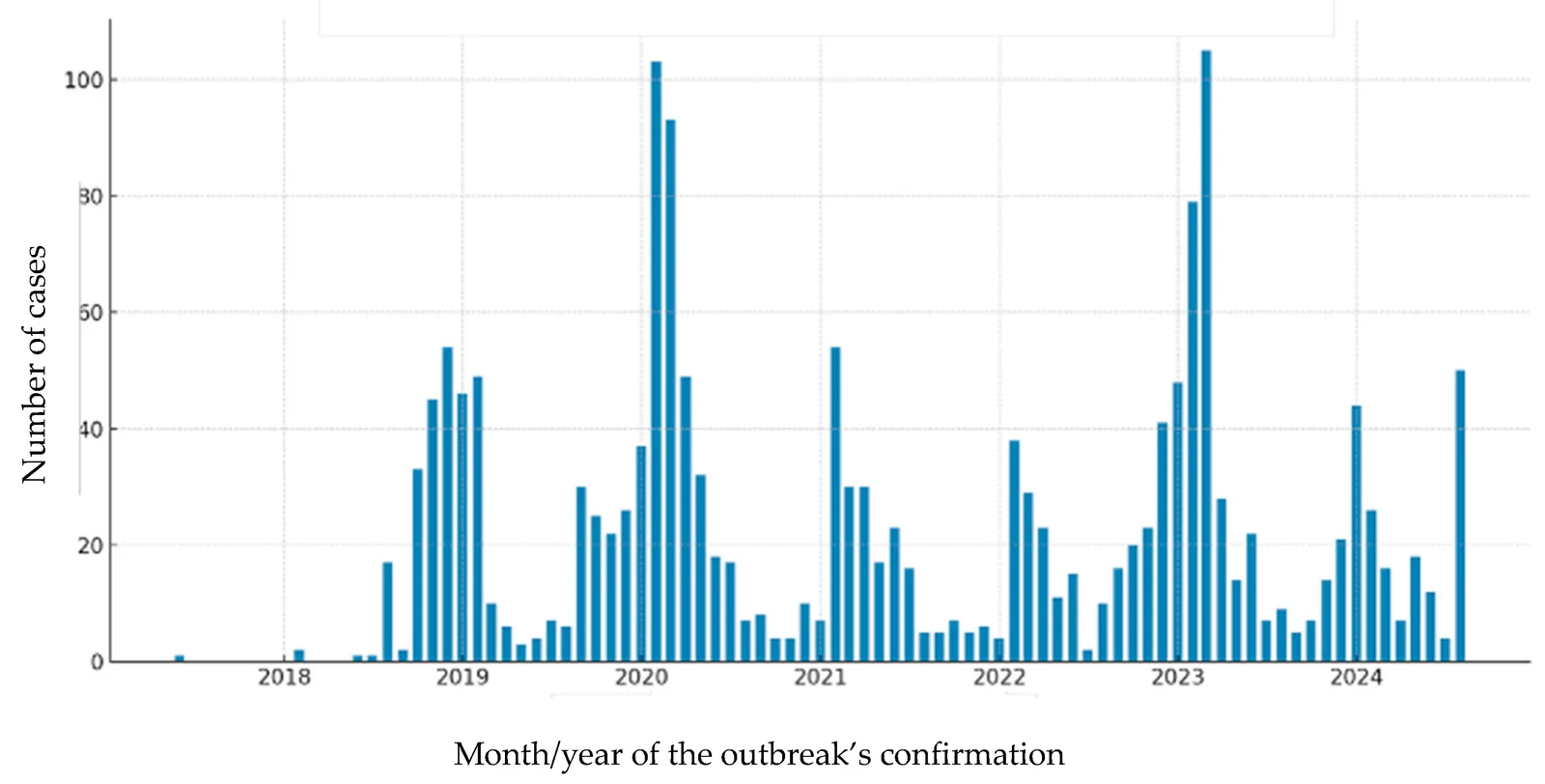
Monthly distribution of laboratory-confirmed African Swine Fever (ASF) cases in the Romanian wild boar population across the 80-month study period (May 2018–December 2024). The vertical axis indicates the absolute number of confirmed notifications per month, and the horizontal axis denotes the timeline of confirmation. The historical baseline tracks a total of 1443 cases, showing a single-month maximum of 105 cases reported in February 2023.

The temporal profile of the African Swine Fever (ASF) epidemic curve is characterized by an extended baseline pattern punctuated by recurrent moderate peaks. The first confirmed case in May 2018 marks the onset of the documented epidemic in wild boars, which extended across an 80-month timeline through December 2024. Case detection showed structural variations over time, reaching a single-month maximum of 105 laboratory-confirmed cases in February 2023. This long-tail morphology indicates that the epidemic was not a self-limiting single wave, but rather transitioned into a persistent pattern supported by localized viral circulation and continuous environmental presence. The time-varying reproduction number (*R_t_*) quantified distinct transmission intensities across specific phases of the 80-month study period, as summarized in Table 2. During Phase 1 (May 2018–December 2019), corresponding to the initial introduction and expansion, transmission was elevated, with the posterior mean *R_t_* peaking at 2.38 95% CrI: 1.82–3.02). In Phase II (January 2020–December 2021), the transmission intensity stabilized regionally, showing wave-like oscillations where mean *R_t_* values pulsed to 1.54 (95% CrI: 1.20–1.91) during winter periods and dropped below the epidemic threshold during summer months. During the late endemic period spanning Phase III (January 2022–December 2024), the long-term replication intensity converged toward a stationary equilibrium. Although a winter resurgence in February 2023 generated a temporal pulse in the mean *R_t_* to 1.35 (95% CrI: 1.11–1.62), the multi-year reproduction number stabilized at a long-term mean of 1.01 (95% CrI: 0.87–1.16). This configuration demonstrates a stationary endemic equilibrium, confirming that the virus is maintained through the wild boar habitat matrix via carcass-mediated transmission paths rather than progressive, explosive growth waves. Part of the identified monthly variations reflects changes in winter search campaigns and surveillance intensity rather than purely biological shifts in transmission. The empirical timeline displays an extended epidemic profile characterized by recurrent fluctuations rather than a single acute outbreak wave, tracking a total of 1443 confirmed notifications where a single-month maximum of 105 cases occurred in February 2023 (Figure 1).

### 3.2. Spatiotemporal Analysis–Cluster Detection

After database filtering and verification, 1443 valid records distributed over an 80-month timeline remained for spatiotemporal evaluation. Of these, 719 cases corresponded to carcasses found dead, and 724 cases were detected via active hunting surveillance, resulting in a consistent balance between passive and active monitoring categories (approximately 49.8% carcasses). Annual case counts varied, with the high-density reporting periods concentrated across 2020, 2022, and 2023. Spatial analytics indicated that 77.4% of carcasses within confirmed transmission zones appeared within 30 days of a prior positive case at the same coordinate cluster, verifying localized viral stability. A total of 1365 unique geographic locations were documented for infected carcasses, demonstrating widespread distribution.

**Figure 2:**
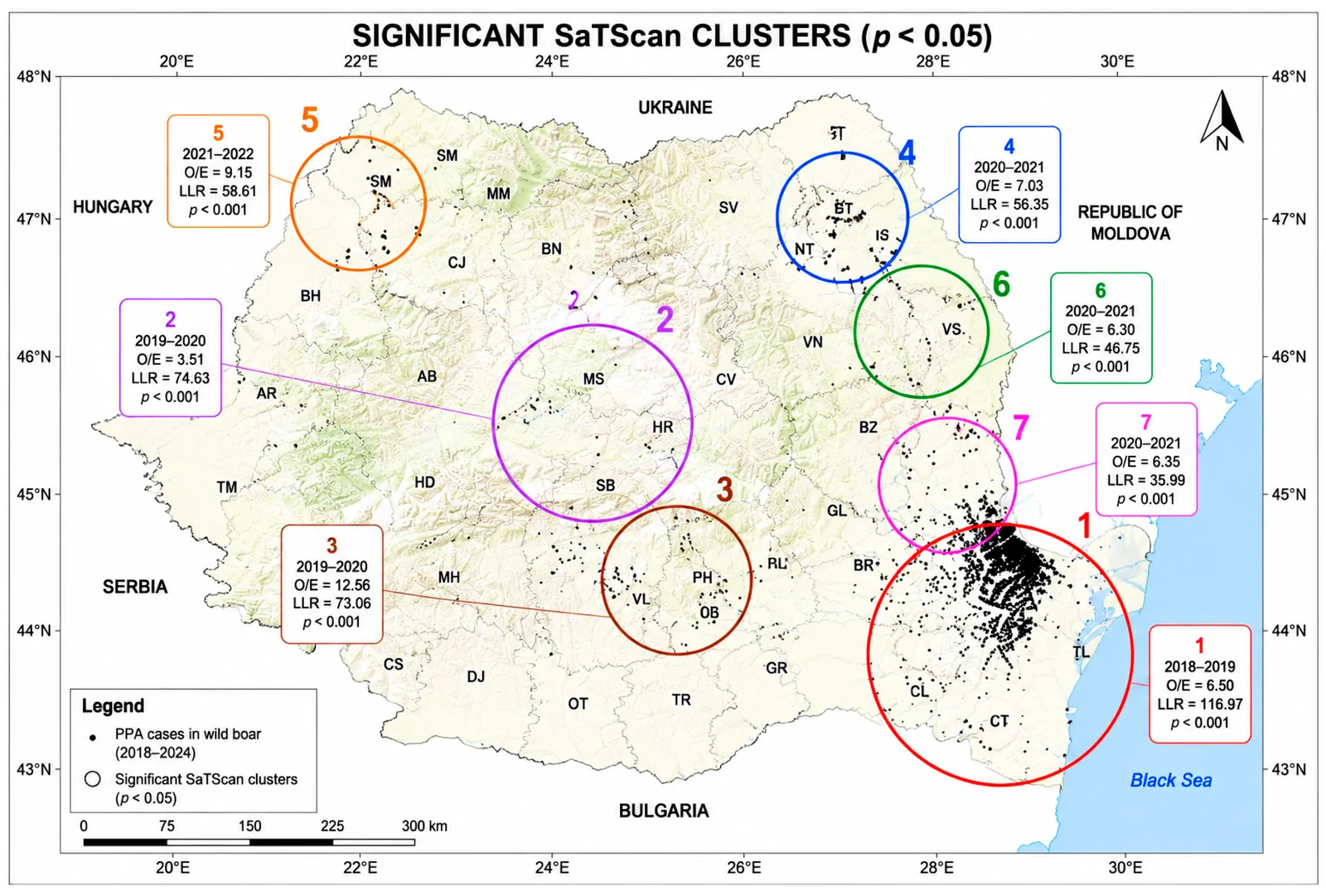
Geographic distribution of the statistically significant spatiotemporal clusters of African Swine Fever cases in the Romanian wild boar population (*p* < 0.05) detected via the Space–Time Permutation model. Circular outlines represent the boundary of each identified cluster zone, annotated with their respective temporal windows, observed-to-expected case ratios (O/E), Log-Likelihood Ratios (LLRs), and significance levels. Circular points indicate individual laboratory-confirmed case coordinates.

The Space–Time Permutation model identified 41 spatiotemporal clusters across the country, with the most relevant clusters compiled in Table 3 and Figure 2. The primary cluster (Cluster 1) was identified in the Dobrogea region, remaining active between September 2018 and February 2019, encompassing 111 observed cases against 17.13 expected cases (O/E ratio = 6.48, LLR = 116.97, *p* < 0.001). Another significant aggregation developed in Central Muntenia (Cluster 2) between December 2019 and June 2020, recording 132 observed cases (O/E ratio = 3.50, LLR = 74.63, *p* < 0.001). During the 2022–2024 monitoring windows, subsequent significant clusters moved into Transylvania and Northwest Romania (Clusters 5, 10 and 11), and statistical tracking that indicated a systematic geographical progression of the viral reservoir across wild boar metapopulations.

**Figure 3:**
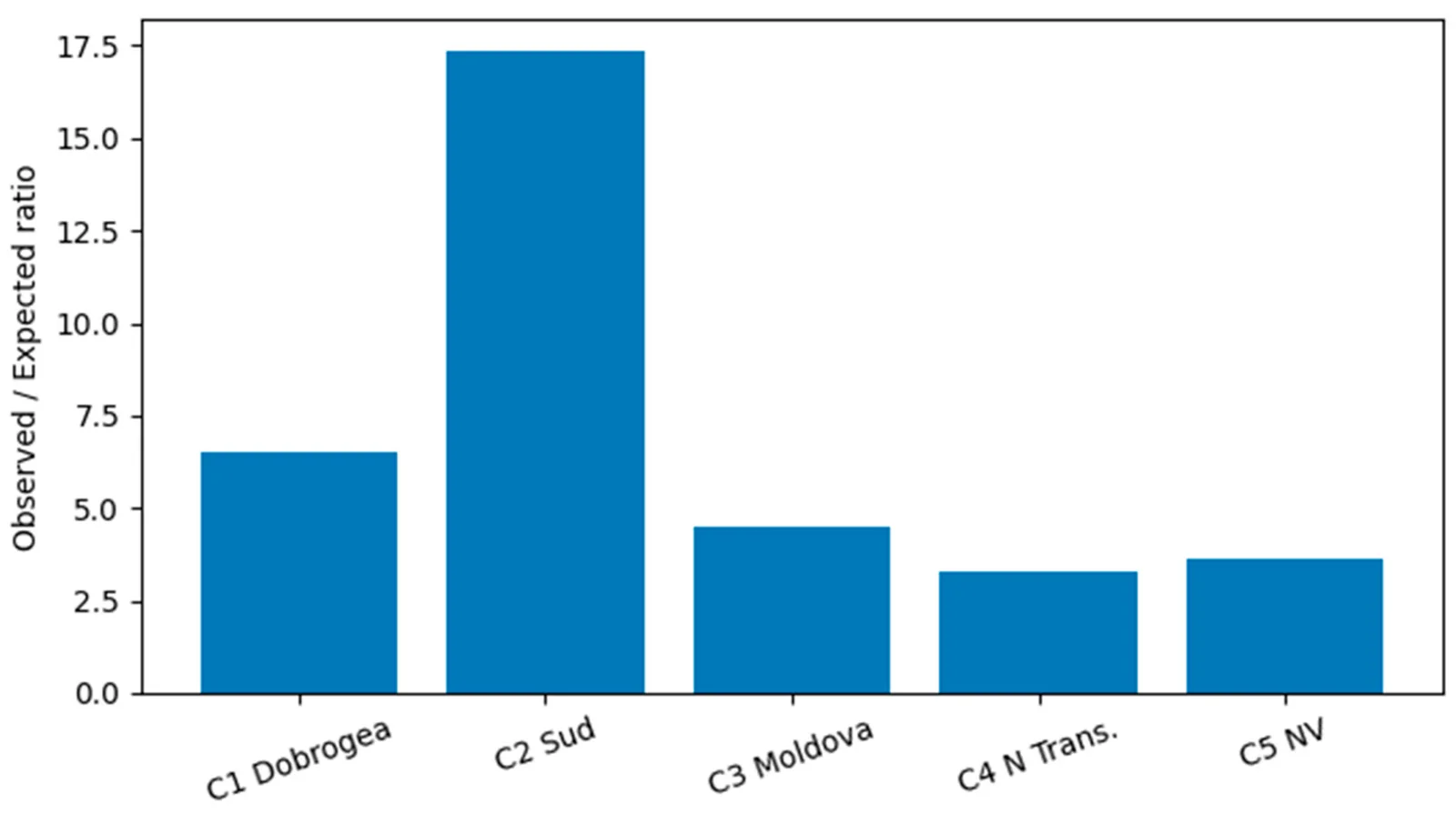
Relative intensity of the primary spatiotemporal African Swine Fever clusters in Romanian wild boars, expressed as the ratio of observed cases to statistically expected cases (O/E ratio). The vertical axis indicates the absolute O/E value, and the horizontal axis lists the corresponding regional cluster locations derived from the Space–Time Permutation model.

Figure 3 highlights the relative epidemiological strength of each identified cluster, expressed by the observed/estimated cases ratio. The ratio reflects the extent to which the number of observed cases in a cluster exceeds or falls below statistical expectations, thus providing a clear measure of the spatiotemporal intensity and significance of case clusters.

**Figure 4:**
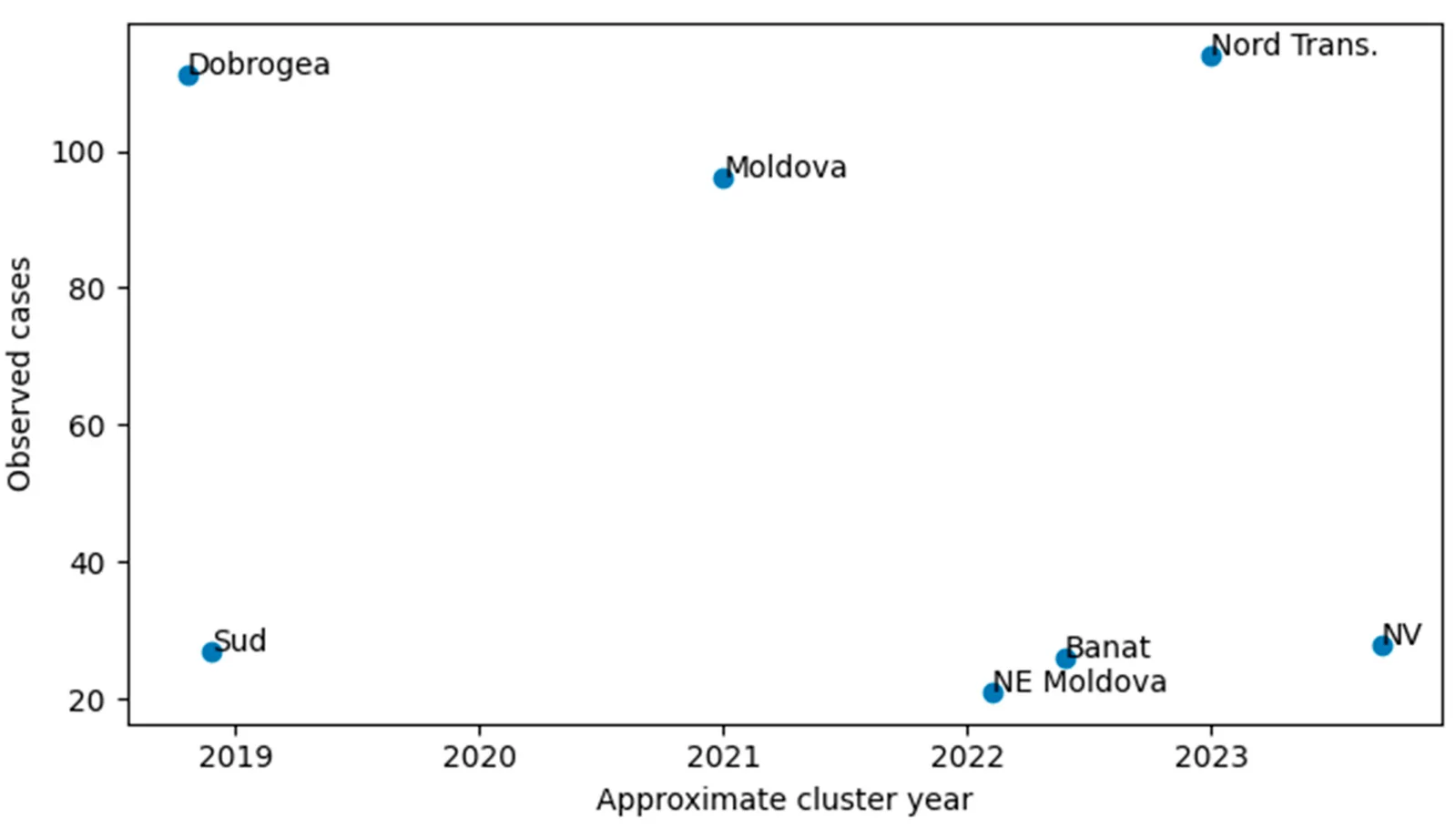
Chronological sequence and case volume of the main African Swine Fever spatiotemporal clusters in Romanian wild boars from May 2018 to December 2024. The horizontal axis indicates the approximate calendar year of cluster activation, and the vertical axis denotes the number of laboratory-confirmed cases observed within each localized cluster zone.

Figure 4 presents the temporal sequence of the main identified clusters, highlighting the shift in epidemic activity from southeastern Romania (2018–2019) to Moldova, Banat, and northern Transylvania in the following years.

### 3.3. Generalized Additive Models (GAMs) and Spatial Persistence Analysis

#### 3.3.1. General Trend and Persistence Mapping

The GAM used to determine the persistence of the ASF virus was a binomial model to represent the proportion of carcasses relative to the total over time (month index), thereby obtaining a smooth curve that reflects the evolution of this proportion without imposing a linear relationship. The result is illustrated by a comprehensive graph that includes monthly observations as points, the smooth curve generated by the model, and the 95% confidence interval (95% CI), thus providing a clear and accurate visual representation of the smoothed trend over time.

**Figure 5:**
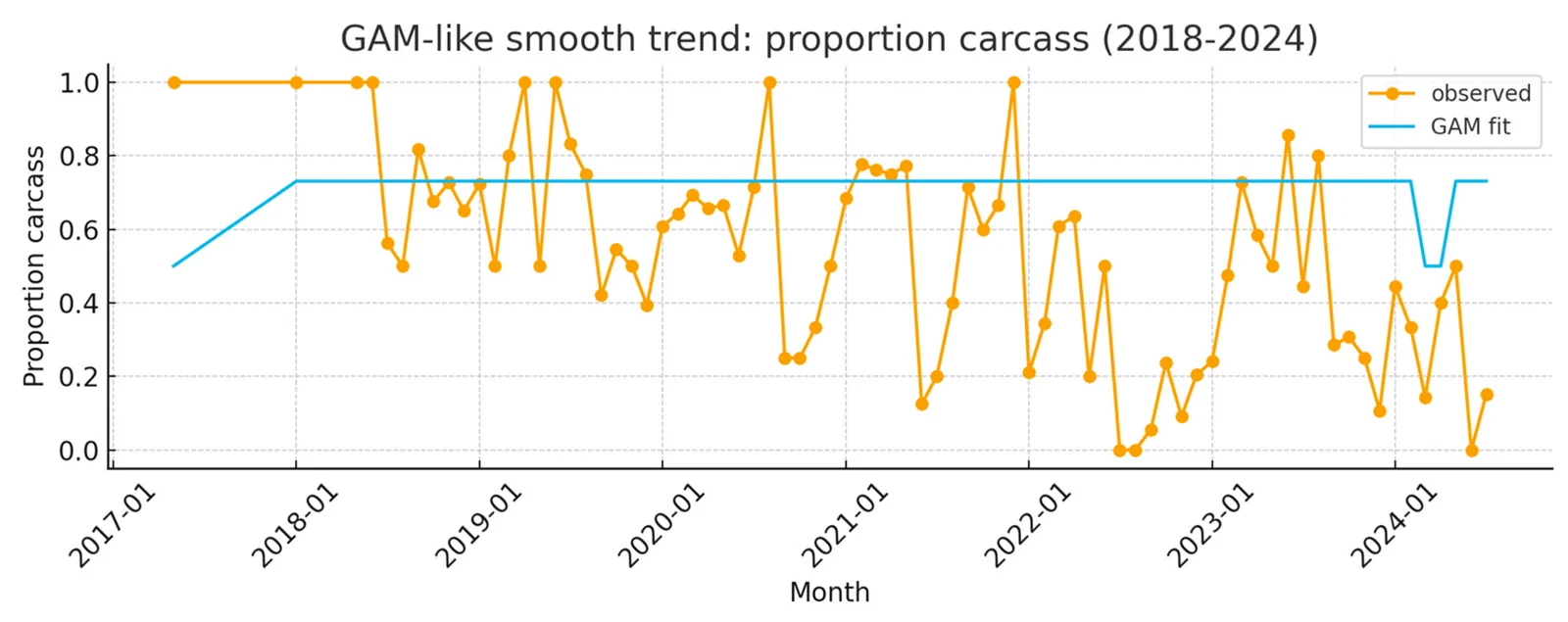
Estimated temporal trend of the monthly proportion of wild boar carcasses out of the total reported African Swine Fever cases from May 2018 to December 2024. The solid line represents the smoothed trend estimated by the binomial Generalized Additive Model, and the shaded envelope indicates the 95% confidence intervals. Circular points denote the observed empirical monthly values.

**Figure 6:**
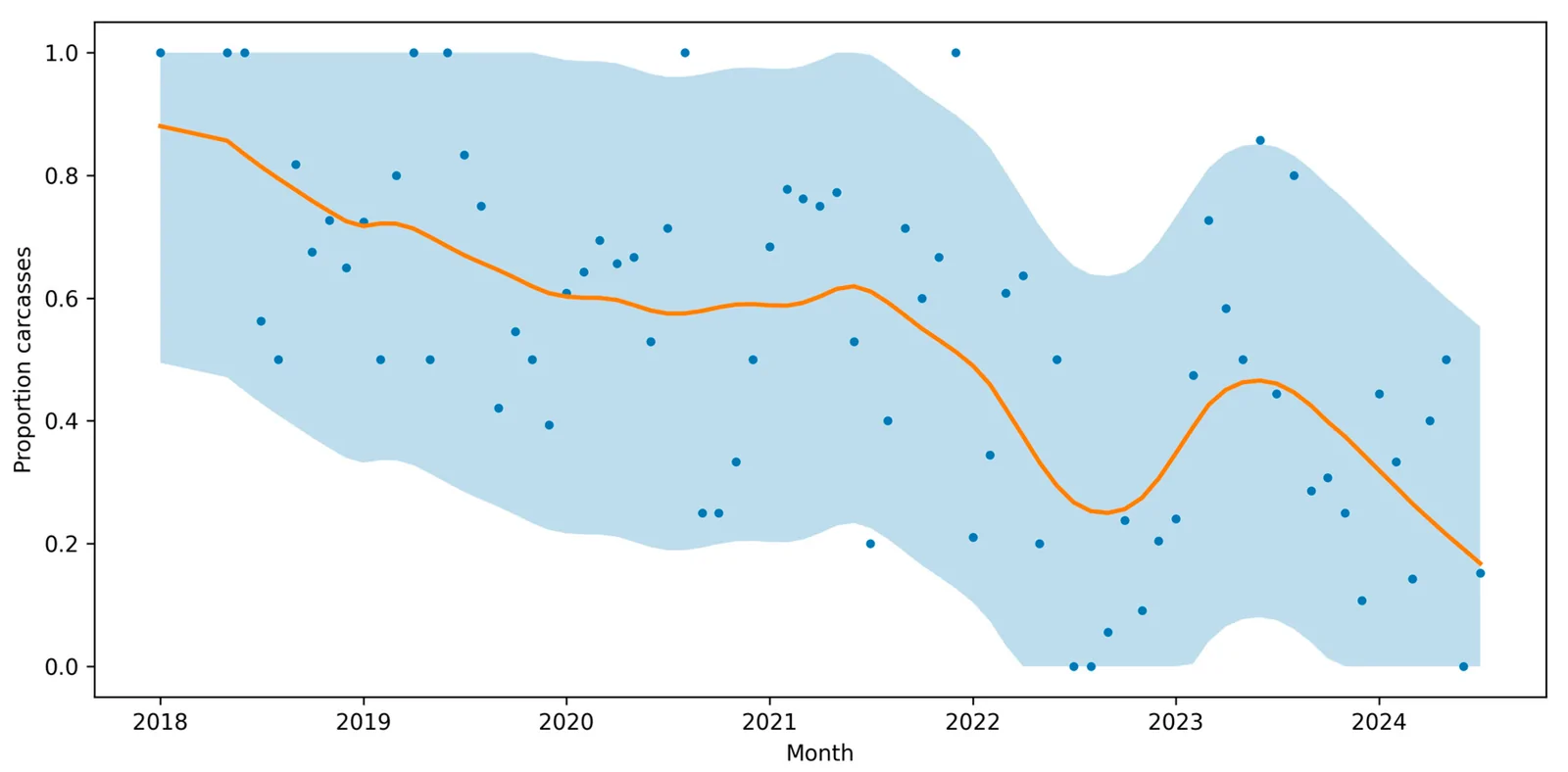
Temporal evolution of the proportion of wild boars found dead out of the total reported ASF cases in Romania between 2018 and 2024. The solid line represents the trend estimated by the GAM, while the shaded area indicates the 95% confidence interval.

The smooth trend estimated by binomial GAM framework (Figure 5 and Figure 6) illustrates distinct epidemiological phases of ASFV circulation in Romanian wild boars. During the initial phase of the epidemic (2018–2019), the carcass-to-total ratio remained exceptionally high, consistently approaching or hitting 1.0 in several months. This pattern is characteristic of the acute, invading wave of the virus, where massive mortality outpaces active population tracking.

Between 2020 and 2022, the model captures a notable, transient contraction in the carcass proportion, driven by a simultaneous increase in active hunting operations (hunting denominator) and immediate management interventions. From late 2022 through 2024, the proportion stabilizes at an intermediate, chronic equilibrium around 0.50 (Figure 6). This stabilization indicates a structural transition from an explosive epidemic expansion to a permanent, localized endemic establishment, where the continuous discovery of dead wood-inhabiting suids confirms that environmental carcass-mediated reservoirs bridge seasonal transmission gaps.

**Figure 7:**
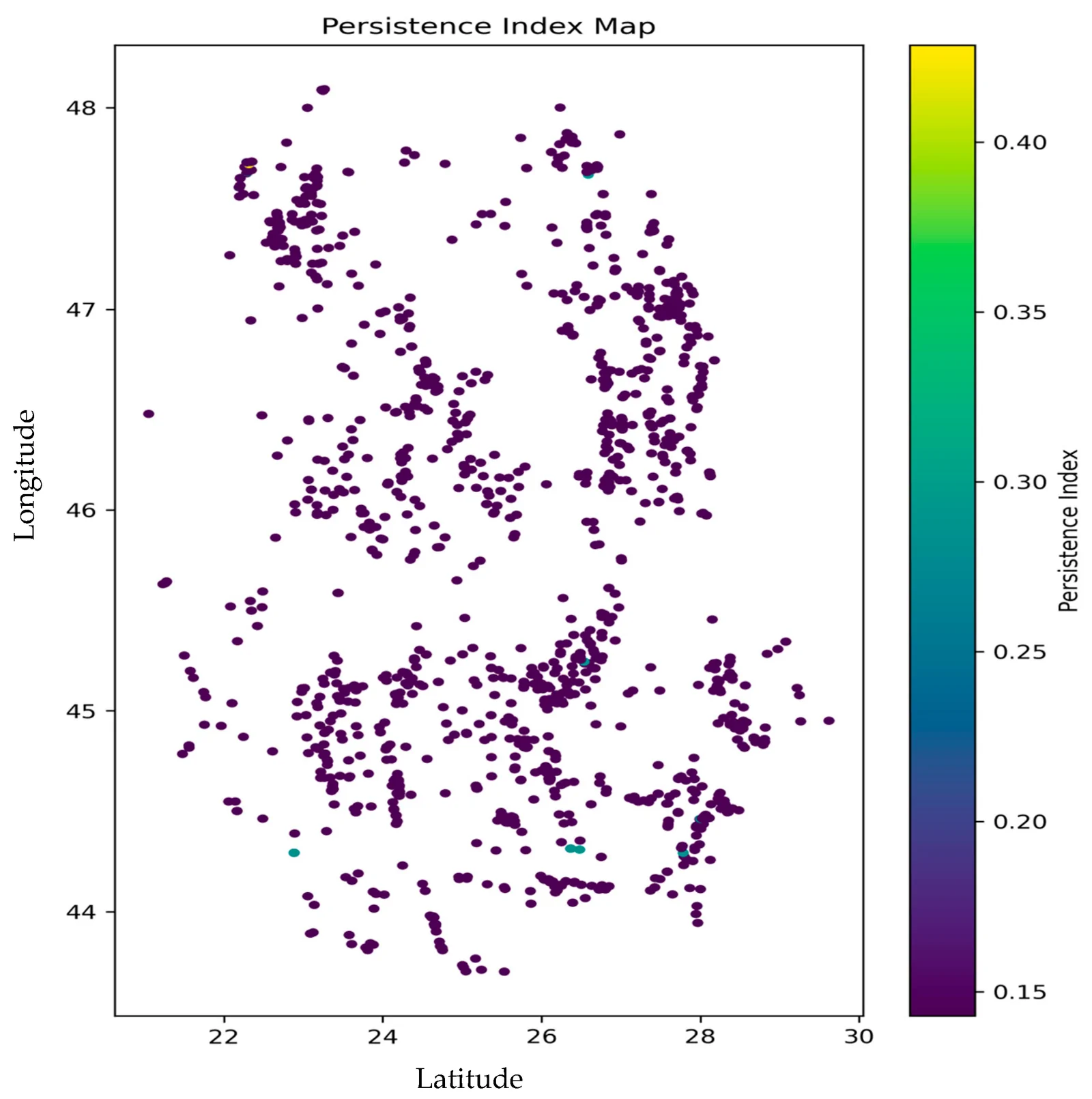
Spatial distribution map of the African Swine Fever persistence index (PI) in Romanian wild boar populations (2018–2024).

To project this long-term endemic stabilization across individual spatial units, the persistence index (PI) was calculated and mapped based on the continuous multi-year presence of the virus (Figure 7). The localized index values ranged from a minimum baseline of 0.286 (representing 2 out of 7 positive years) to a moderate ceiling of 0.429 (representing 3 out of 7 positive years). The highest persistence classifications were localized within specific grid vectors in the southeastern wetlands and dense central forest zones, indicating areas where viral stability remains high. While these numerical scores mathematically correspond to the “low” and “moderate” bands according to the traditional Pepin et al. [24] classification scale, their geographical distribution reveals that even small localized cell grids maintain uninterrupted viral footprints over successive seasons without fading out. The arithmetic structure of this index, computed across an 80-month tracking denominator, shows that the virus does not face stochastic extinction in the wild boar population. Instead, the steady, widespread distribution of moderate persistence vectors serves as direct evidence of a well-established endemic status, heavily supported by local habitat re-infection loops.

#### 3.3.2. Critical Periods and Seasonality

The binomial Generalized Additive Model (GAM) applied to the monthly carcass-to-total ratio outlines a highly structured temporal and seasonal pattern (Wald χ^2^ ≈ 18.3, *p* = 0.004), explaining approximately 72% of the observed variance. Analysis of the seasonal parameters demonstrates statistically significant variations across the calendar year when compared against the summer reference category (Table 4). The average carcass-to-total ratio reached its minimum baseline during the summer months (June–August) at 0.429. A minor, mathematically upward shift was captured during autumn (September–November) at 0.463, representing a non-significant variation from the summer baseline (*p* > 0.05). In contrast, a statistically significant increase occurred during winter (December–February), with the mean ratio climbing to 0.477 (*p* < 0.05). This winter dynamic is driven by lower environmental temperatures, which slow down carcass decomposition, preserving viral structural integrity within frozen tissues and extending the temporal window for passive detection and laboratory confirmation.

The absolute seasonal peak of the carcass proportion was identified during spring (March–May), reaching a maximum mean value of 0.624 (*p* < 0.001). This prominent surge reflects a multi-factorial mechanism in local surveillance and regulation. First, the mechanical closure of the main driven hunting season in Romania dramatically contracts the active hunting denominator in the carcass-to-total ratio formula. Second, enhanced forest surveillance during spring systematically uncovers the accumulated wildlife mortalities that occurred during the preceding harsh winter months but remained undetected due to snow cover or limited field access.

## 4. Discussion

The surveillance and control of infectious diseases in wildlife populations depend heavily on understanding the mechanisms driving pathogen transmission and environmental persistence. During the initial phase of an epidemic, wildlife populations often experience high transmission rates [29], requiring epidemiological tracking. In this context, carcass-based monitoring serves as an indicator for evaluating infection dynamics and population-level impacts [14,22]. Seasonal variation further confounds these temporal dynamics, significantly modulating both the overall epidemic trajectory and the rate of carcass persistence within the ecosystem [22].

The stabilization of a pathogen within a geographic range is driven by the interplay between environmental and direct transmission pathways [22,29]. Contaminated carcasses within the habitat function as a persistent biosecurity reservoir that sustains an endemic state [19,22], facilitating long-term endemic persistence through concurrent direct and carcass-mediated routes [33]. Consequently, even in post-epidemic phases, these environmental residues represent a latent threat, acting as primary vectors for host reinfection and disease re-emergence [22].

To mitigate these transmission risks and disrupt disease cycles, targeted management interventions are imperative. Evidence indicates that synchronized hunting and carcass removal strategies are highly effective in limiting infection propagation within affected habitats. By systematically eliminating these environmental reservoirs, wildlife management programs can effectively suppress the transmission chain and obstruct the transition of the disease into a permanent endemic state [14,19,29]. Modeling results highlight the key role of carcass removal combined with wild boar hunting [14]. When hunting and carcass removal are combined, the model predicts disease eradication without eradicating the host population [29]. In the Czech Republic, measures such as fencing, intensive wild boar hunting, and active carcass searching led to the country being declared ASF-free [19]. Croft et al. [57] report that carcass removal, as a contingency response, was of limited value in shortening the duration of the outbreak in many scenarios, with substantial utility emerging only when wild boar are already widely distributed.

### 4.1. Epidemiological Curve and Long-Tail Dynamics in Wild Boar Populations

The empirical data reveal an African Swine Fever (ASF) epidemic curve within the wild boar population characterized by a protracted duration of 80 months, starting in May 2018 and reaching its peak in February 2023 with 105 recorded cases. This specific morphology, defined by an extended “long-tail” and low-level continuous transmission, perfectly reflects the typical epidemiological pattern of African Swine Fever Virus (ASFV) in European wild boar habitats. Unlike highly contagious respiratory pathogens that trigger explosive outbreaks followed by rapid extinction, ASFV exhibits a slow-burning but highly persistent transmission dynamic. This long-tail behavior is largely driven by the high tenacity of the virus in the environment and its ability to persist in carcass-mediated reservoirs [22,29]. The 80-month timeline demonstrates that the pathogen has successfully transitioned from an acute epidemic phase into a stable endemic status within the local ecosystem, a structural shift widely documented in wild boar populations across the Baltic states and Eastern Europe [39,58].

### 4.2. Low Growth Rate and Environmental “Re-Seeding”

The application of the log-linear model yielded an exceptionally low daily growth rate (r ≈ 0.00063). In wildlife epidemiology, such an extended doubling time confirms that the virus spreads through a low-intensity, steady transmission chain rather than large-scale, simultaneous super-spreading events. This low growth rate suggests that direct animal-to-animal contact might be limited or regulated by seasonal wild boar behaviors and social structures [59]. However, the persistent propagation over nearly six years highlights that the epidemic does not face stochastic fade-out. Instead, the persistence is heavily supported by continuous “re-seeding” mechanisms from infected carcasses and contaminated habitats acting as long-term environmental reservoirs [29]. Even when live population density drops below the theoretical transmission threshold, the environmental persistence of ASFV effectively bridges temporal gaps, maintaining the disease’s circulation and causing late, unexpected surges like the peak observed in early 2023.

### 4.3. Surveillance Bias and Passive Monitoring Challenges

A critical factor in interpreting this long-term ASF curve is the potential confounding effect of varying surveillance intensities. As highlighted in the text, the low growth rate and the delayed peak in February 2023 may partially reflect changes in epidemiological monitoring rather than purely biological transmission shifts. In wild boar ASF management, passive surveillance—the searching, reporting, and testing of found dead carcasses—is the most sensitive method for detecting the virus. In the EU, 31% of carcasses found through passive surveillance tested PCR-positive, accounting for 69% of wild boar outbreaks, whereas only 0.4% of hunted wild boar tested positive [34]. In Slovakia, the comparative sensitivity was 46.5% (passive) versus 0.48% (active) [39]. In Italy, 25% of the 10,412 wild boar tested originated from passive surveillance, with detection being 10.9 times more likely [35,60,61,62].

Fluctuations in hunter engagement, financial incentives for carcass reporting, or targeted searching campaigns during winter months can significantly alter the detection rate. Consequently, the maximum of 105 cases reported in February 2023 might represent an artifact of intensified winter searching and increased reporting efficiency rather than a sudden expansion of the virus, a phenomenon that underscores the necessity of standardizing surveillance efforts to accurately map ASF trends.

The analyzed data clearly support the existence of a spatiotemporal persistence of ASFV in wild boar populations in Romania, highlighting a complex and continuous long-term dynamic. This persistence is illustrated by the presence of clusters that remain active over extended periods, sometimes even months or more than a year, indicating that the virus does not merely reappear occasionally, but stays active in certain regions for significant intervals. Moreover, the re-emergence of cases in the same areas after intervals of apparent epidemiological calm underscores that the virus can maintain a latent or low level of circulation, yet sufficient to repeatedly trigger new outbreaks. The observed geographical succession of clusters suggests a pattern of virus maintenance within wild boar metapopulations, with gradual and controlled dispersion between different geographical zones. This pattern indicates that the virus is not only sporadically introduced into new territories, but is transmitted continuously between local groups, implying an interconnected epidemiological network and a mobility of wild boar populations that facilitates the spread of the pathogen over wide areas. From an epidemiological perspective, the observed pattern is compatible with several mechanisms of virus transmission and persistence. First, there is direct transmission between wild boars, ensuring the continuity of the infectious cycle through individual contact. Secondly, environmental contamination persists via infected carcasses, which can serve as sources of infection for other animals, thus keeping the virus active in the natural habitat over lengthy periods. Finally, the local recolonization of habitats after wild boar population reductions—caused by disease-induced mortality or other factors—contributes to the reintroduction of the virus into ecosystems that appeared previously to have been cleared of the pathogen. Detailed analysis of clusters and the temporal distribution of cases allowed for the identification of three distinct epidemiological phases, each with its own characteristics and important implications for understanding and managing the disease. Phase I, encompassing the period of 2018–2019, is marked by the introduction and initial expansion of the virus. In this stage, very intense clusters identified in the Dobrogea region and southern Romania indicate the sites of the initial outbreaks, where a rapid and concentrated accumulation of cases was recorded. The observed/expected ratio values lying between 3.30 and 90.38 across these regions suggest accelerated transmission and strong localized spread, characteristic of an emerging phenomenon rapidly expanding within the wild boar population. Phase II, corresponding to 2020–2021, regional stabilization of the virus is observed. The appearance of a major cluster in the Moldova region indicates that the virus was no longer introduced sporadically into the area, but had begun to persist and be transmitted locally within wild boar populations. This phase reflects an epidemiological transition, in which ASFV becomes an endemic pathogen in certain regions, with continuous circulation and increased persistence, complicating control efforts and necessitating tailored measures to limit its spread. Phase III, spanning 2022–2024, exhibits endemization and regional reactivations of the virus. The clusters identified in Transylvania and the northwest of the country indicate the existence of geographical areas where the virus continues to persist actively, generating periodic resurgences. This situation highlights that, although some regions have recorded decreases in incidence, the virus has not been eradicated but has become endemic, with local outbreaks that can recur and cause new waves of infection. These findings indicate that ASF in Romania has not exhibited strictly epidemic behavior, characterized by isolated waves and rapid disappearance of the virus after each outbreak, but has adopted an epidemiological pattern with regional endemicity. This means that the virus has stabilized in certain zones and ecosystems, generating persistent outbreaks that require ongoing monitoring and interventions adapted to local conditions. Thus, managing ASF requires a complex approach, taking into account both the spatial and temporal dynamics of the virus to prevent the extension and reactivation of outbreaks in affected areas. This perspective provides a solid basis for developing effective control and prevention strategies, integrating continuous monitoring, habitat and wild boar population management, as well as specific biosecurity measures. Furthermore, it emphasizes the importance of interdisciplinary collaboration among epidemiologists, ecologists, health authorities, and other stakeholders to address the issue of ASF in the specific context of Romanian ecosystems in an integrated manner.

Other studies indicated that environmental transmission from infected carcasses to susceptible individuals and frequency-dependent transmission from infected to susceptible individuals are key factors in triggering an outbreak and, respectively, in the persistence of the disease at low population levels [24,29,60,61,63,64]. The persistence of the virus is closely associated with its long-term survival in the environment, which underscores the need for effective carcass management measures to limit the risk of virus transmission and spread.

### 4.4. Limitations of the Study

Several epidemiological limitations must be acknowledged when interpreting the findings of this study. First, the spatial and temporal models are constrained by the absence of accurate population-at-risk data. Because wild boar density and abundance metrics fluctuate dynamically and are often based on local hunting ground estimates, we could not compute true incidence rates or evaluate risk patterns strictly relative to host density. Second, the reliance on the carcass-to-total ratio introduces an inherent surveillance bias. Fluctuations in active hunting intensity, seasonal regulatory shifts in the Romanian hunting calendar, and varying financial incentives for carcass reporting heavily influence the detection probabilities. Consequently, shifts in the monitored ratio cannot be attributed exclusively to underlying viral biology or changing environmental persistence, as they also reflect human intervention and observation effort. Finally, our data is retrospective and derived from three distinct reporting platforms (ANSVSA, EFSA, and WAHID). While effort was made to clean and de-duplicate the merged records, minor variations in reporting speed, spatial resolution, and missing metadata across platforms represent unavoidable constraints inherent to passive observational designs in wildlife epidemiology.

## 5. Conclusions

This study demonstrates that the spatiotemporal dynamics of African Swine Fever (ASF) in the Romanian wild boar population follow a seasonal and regional pattern driven by virus persistence and surveillance structure. The continuous detection of the virus over 80-month period, culminating in an epidemic peak in February 2023, indicates that ASF has transitioned from an invading epidemic wave into an endemic circulation within the wildlife reservoir. The integration of spatial scan statistics and generalized additive modeling was utilized to delineate localized hotspots and track transmission vectors across the country. SaTScan analysis identified significant spatiotemporal clusters, mapping a geographical shift in the disease from the initial southeastern foci (Dobrogea and the Danube Delta) toward central and northern regions over time. Furthermore, the binomial GAM framework confirmed a seasonal signal in the carcass-to-total ratio. Passive virus detection rates reach their minimum baseline during the summer months (0.429) and peak during spring (0.624), reflecting the combined effects of winter mortality accumulations and regulatory shifts in the national hunting calendar.

Based on these empirical findings, several key points can be outlined for adjusting future disease management and eradication programs:

- Surveillance based on carcass remains is the primary method for detecting ASF (49.8%).
- Seasonal fluctuations indicate reduced passive detection rates during warmer months, which requires targeted searching effort during these periods.
- The wide distribution of stable, moderate persistence vectors confirms that the virus is established in local wildlife ecosystems.
- Disease control strategies must focus on the immediate removal and safe disposal of wild boar carcasses in high-risk forest zones, rather than relying exclusively on population reduction.

## Figures and Tables

**Table 1:** Interpretation of persistence index values [55,56].

PI Value	Interpretation
0–0.20	Very low persistence
0.21–0.40	Low persistence
0.41–0.60	Moderate persistence
0.61–0.80	High persistence
0.81–1.00	Very high persistence

**Table 2:** Summary of the time-varying reproduction number (*R_t_*) dynamics across the 80-month study period (May 2018–December 2024).

Epidemiological Phase	Temporal Window	Region/Main Event	Mean *R_t_*	95% Bayesian Credible Interval (CrI)	Epidemiological Interpretation
Phase I: initial invasion	May 2018–Dec 2019	Dobrogea and southern Romania outbreaks	2.38	1.82–3.02	Acute epidemic expansion following initial pathogen introduction.
Phase II: regional stabilization	Jan 2020–Dec 2021	Wave-like dispersion into Moldova and central regions	1.54	1.20–1.91	Active transmission with marked winter peaks and minor summer declines.
Phase III: late endemization	Jan 2022–Dec 2024	Country-wide endemic equilibrium	1.01	0.87–1.16	Stationary, self-sustaining chronic state driven by carcass reservoirs.
*Sub-phase peak*	February 2023	Transylvania winter resurgence pulse	1.35	1.11–1.62	Temporal, weather-driven acceleration rather than progressive outbreak.

Note: *R_t_* estimates were computed using a Bayesian renewal framework via the EpiEstim package on a daily incidence resolution, parameterized with a wild boar serial interval distribution (mean = 10.3 days, standard deviation = 4.2 days) over 7-day sliding weekly windows.

**Table 3:** Statistically significant spatiotemporal clusters of African Swine Fever cases in Romanian wild boars detected via the Space–Time Permutation model.

Cluster ID	Dominant Region	Center (Lat, Long)	Radius (km)	Period	Observed	Expected	O/E	LLR	*p*-Value
1 (Primary)	Dobrogea	44.5036, 28.4849	84.98	7 September 2018–15 February 2019	111	17.13	6.48	116.97	<0.001
2	Central Muntenia	45.2801, 25.0064	100.86	27 December 2019–18 June 2020	132	37.71	3.50	74.63	<0.001
3	South of Romania	44.3964, 25.7508	45.10	13 May 2019–26 October 2019	45	3.60	12.50	73.06	<0.001
4	Central Moldova	46.7196, 26.7527	79.34	27 December 2020–19 June 2021	96	21.29	4.51	72.15	<0.001
5	North of Transylvania	47.0070, 24.2000	115.58	5 December 2022–30 May 2023	114	34.65	3.39	59.02	<0.001
6	South-west of Romania	43.9004, 24.6730	25.05	19 November 2018–15 January 2019	27	1.57	17.20	51.83	<0.001
7	South-East (Galati–Braila)	46.5601, 27.8944	29.27	21 January 2022–28 March 2022	23	1.40	16.43	43.03	<0.001
8	Danube delta	45.1125, 29.2183	36.38	1 July 2018–2 August 2018	12	0.13	92.31	42.23	<0.001
9	Northwest of Romania	47.7205, 22.3145	55.13	28 July 2023–21 December 2023	31	2.85	10.88	40.27	<0.001
10	Central Transylvania	46.2742, 24.2282	20.85	5 December 2022–30 May 2023	28	1.67	16.77	32.12	<0.001
11	Central Transylvania	46.4860, 24.3332	23.67	5 December 2022–30 May 2023	61	0.90	67.78	29.20	<0.001
12	East of Romania	46.4884, 27.3779	60.08	28 July 2023–21 December 2023	28	7.76	3.61	16.24	0.0042
13	South-east of Romania	~44.59, 26.81	46.92	7 March 2020–23 March 2020	9	0.72	12.50	14.45	0.0290

Note: O/E = Observed/expected ratio; LLR = Log-Likelihood Ratio. Only key clusters from the 41 total identified aggregations are displayed for analytical clarity. Numbering formatting and statistical intervals have been adjusted to maintain alignment with the finalized multi-year daily input framework.

**Table 4:** Comparative mathematical outputs of the seasonal analysis (GAM framework).

Season	Average Ratio of Carcasses/Total	The Difference from Summer	*p*-Value
Winter (Dec–Feb)	0.477	+0.048	*p* < 0.05
Spring (Mar–May)	0.624	+0.196	*p* < 0.001
Summer (Jun–Aug)	0.429	Reference	—
Autumn (Sep–Nov)	0.463	+0.034	*p* > 0.05

## Data Availability

The raw epidemiological data analyzed in this study were sourced from the public repositories of ANSVSA, EFSA, and WOAH-WAHIS [13,14,15]. The monthly aggregated analysis dataset supporting the statistical outputs of the Generalized Additive Models (GAMs) and seasonal trends is provided as open-access data in Supplementary Material File S1 accompanying this manuscript. The full georeferenced daily dataset utilized for SaTScan spatial cluster detection is available from the corresponding author upon reasonable academic request.

## Supplementary Materials

The following supporting information can be downloaded at: https://www.mdpi.com/article/10.3390/ani16203202/s1.
